# Dietary *β*-Mannanase Affects the Growth, Antioxidant, and Immunes Responses of African Catfish, *Clarias gariepinus*, and Its Challenge Against *Aeromonas hydrophila* Infection

**DOI:** 10.1155/2024/5263495

**Published:** 2024-10-28

**Authors:** Ibrahim Adeshina, Bilal Ahamad Paray, Eijaz Ahmed Bhat, Shahid Sherzada, Olaolu O. Fawole, Dalhatu J. Bawa, Thais Pereira da Cruz, Lateef O. Tiamiyu

**Affiliations:** ^1^School of Aquaculture, National University of Agriculture, Port Nove, Benin; ^2^Department of Aquaculture and Fisheries, University of Ilorin, Ilorin, Nigeria; ^3^Department of Zoology, College of Science, King Saud University, P.O. Box 2455, Riyadh 11451, Saudi Arabia; ^4^Microbiology/Molecular Physiology of Prokaryotes, Institute of Biology II, University of Freiburg, Schänzlestraße 1, Freiburg 79104, Germany; ^5^Department of Zoology, Government College University, Lahore, Pakistan; ^6^Department of Fisheries and Aquaculture, Ladoke Akintola University of Technology, Ogbomoso, Nigeria; ^7^Department of Forestry and Fisheries, Kebbi State University of Science and Technology Aliero, Lagos, Nigeria; ^8^Animal Science Graduate Degree Program, State University of Maringa, Maringa, PR, Brazil

**Keywords:** *β*-Mannanase, African catfish, *A*. *hydrophila*, antioxidant, immunity, fish

## Abstract

One of the most farmed fishes is the African catfish, *Clarias gariepinus*. Its production has increased by 20% annually on average during the last 20 years, but the occurrence of fish diseases, especially bacterial such as *Aeromonas hydrophila* infections, is hindering its activities. Also, the incorporation of plant-derived substances in aquafeeds is limited since they frequently contain different antinutritional factors, like nonstarch polysaccharides (NSPs). However, supplementing fish diets with *β*-mannanase could increase growth, antioxidants, and immunity. Despite the advantage of *β*-mannanase, its effects on growth, digestive enzymes, antioxidants, and immunity in African catfish need to be elucidated. This study examined the effects of dietary β-mannanase on the growth performance, liver enzymes, antioxidant profiles, immunity, and protection of African catfish, *C. gariepinus*, against *A. hydrophila* infection. Five isonitrogenous diets were prepared to have 400 g/kg crude protein and supplemented with *β*-mannanase at 0, 1500, 3000, 4500, or 6000 thermostable endo, 1,4-*β*-mannanase units (TMUs)/kg diet and fed to 300 juveniles of the African catfish, *C. gariepinus* (mean weight 12.1 ± 0.1 g) for 12 weeks. Then, 10 fish from each tank received an intraperitoneal injection of 0.1 mL of *A. hydrophila* (5.0 × 10^5^ CFU/mL) and observed for 14 days. Results showed dietary *β*-mannanase levels considerably improved growth performance but did not affect fish survival. Also, amylase, protease, and lipase levels were significantly promoted in the fish fed with *β*-mannanase-fortified diets than the control group (*p*  < 0.05). Enhanced gut villi and intestinal absorption areas, haematlogical profiles, and liver enzymes but reduced gut viscosity were observed in fish-fed *β*-mannanase-fortified diets (*p*  < 0.05). In a dose-dependent order, including *β*-mannanase in the meals of African catfish raised the levels of glutathione (GSH), glutathione peroxidase (GPx), superoxide dismutase (SOD), glutathione-S-transferase (GST), and glutamate cysteine ligase (GCL) activities and decreased the malondialdehyde (MDA) values in African catfish (*p*  < 0.05). Also, fish immunity was greatly (*p*  < 0.05) enhanced due to supplementation of the diet with *β*-mannanase. In addition, fish-fed diets comprising 6000 TMU *β*-mannanase/kg diet showed the lowest rates of fish mortality (7.5%) (*p*  < 0.05). Therefore, feeding African catfish, *Clarias gariepinus*, *β*-mannanase enhanced growth performance, increased activity of digestive enzymes, gut morphology, enhanced generation of short-chain fatty acids, digesta potential of hydrogen (pH), and improved antioxidant profiles and immunity at the optimum dose of 5800 TMU/kg diet. Additionally, *β*-mannanase protected African catfish against *A. hydrophila* infection.

## 1. Introduction

Aquaculture became one of the most developed sectors as a result of the drastic decline in supplies from captured fisheries brought by, among other things, overfishing, unreported, illegal, and reckless fishing, habitat damage, and pollution. The supply of fish has grown significantly and steadily due to production from aquaculture. African catfish, *C. gariepinus*, is one of the most popular and widely distributed fish species in the world. This species has been tamed and developed for a variety of uses, such as aquaculture. One of the most widely farmed fish species in tropical Africa is African catfish. Africa and Asia are seeing a rapid development in African catfish. Production has increased by 20% annually on average during the last 20 years [1, 2]. Intensive fish farming is considered one of the most effective techniques for achieving higher growth performance and economic returns in fish farming, including African catfish. However, the feasibility of this culture method is compromised by the occurrence of fish diseases, particularly bacterial infections [3, 4]. An outbreak of *A. hydrophila* has been associated with fin rot, haemorrhage, septicemia, furunculosis, and red sore disease in African catfish [5]. These infections cause huge fish deaths and financial losses [6–9]. Therefore, it is crucial to safeguard fish from bacterial infections during commercial production.

According to FAO [1], African catfish is an omnivore fish species that may consume plants. These qualities are beneficial because they limit competition for human resources, which is essential to satisfy the growing need for feedstuffs that are both highly nutritious and sustainable [10]. However, the incorporation of plant-derived substances in aquafeeds is limited since they frequently contain different antinutritional factors, like nonstarch polysaccharides (NSPs) [11, 12]. With 1.3%–2.7% of *β*-mannans, which are mostly made up of galactomannans, glucomannans, and *β*-mannans that fish cannot digest, soybean meal (SBM) is a particularly rich source of NSPs in feed [13]. As the digesta becomes viscous, digestive enzymes are unable to interact with their corresponding substrates due to the increased viscosity [14–16]. As a result, these deficiencies have a detrimental effect on the absorption and digestion of nutrients, which eventually lowers fish development performance [17]. The ability of *β*-mannanase to enhance the growth and feed efficiency of aquatic animals has drawn a lot of interest in recent years [17, 18]. As a result, using *β*-mannanase effectively holds promise for reducing the detrimental impacts of NSPs on aquatic animals' nutrition [11, 14, 19].

The enzyme *β*-mannanase is responsible for catalyzing the hydrolysis of *β*-mannan bonds, thereby reducing the viscosity of digesta [20]. Mannanase aids digestion by hydrolyzing mannan bonds and reducing the viscosity [21]. Specifically, *β*-mannanase reduces the number of potentially hazardous or pathogenic organisms while increasing the proliferation of helpful bacteria, thus modulating the gut microbiota [20]. In a recent study on common carp, it was shown that adding *β*-mannanase to their diet increased the population of beneficial bacteria from the phylum Firmicutes, which led to improved growth. At the same time, the presence of potentially harmful bacteria, such as *A. hydrophila*, was reduced [17]. In the same direction, dietary *β*-mannanase supplementation modulated gut microbiota of Nile tilapia to promote beneficial bacteria such as *proteobacteria*, *Actinobacteria*, and *Firmicutes* species and reduced pathogenic organisms like *Escherichia* species [22].

According to Guan et al. [23] and Tiwari et al. [24], this regulation is caused by *β*-mannanase's breakdown of complex carbohydrates like mannans, which releases fermentable substrates for the gut flora. By changing the pattern of short-chain fatty acid (SCFA) production and reducing the intestinal potential of hydrogen (pH) as a result, such manipulation may also help reduce the number of harmful organisms [25, 26]. According to Hoseinifar, Sun, and Caipang [27], SCFAs such as acetate, propionate, and butyrate are essential for gut health and host metabolism. Research has shown that SCFAs improve intestinal health by increasing the size of villi and serving as a source of energy [28]. Inspite of the advantage of *β*-mannanase, its effects on growth, digestive enzymes, antioxidants, and immunity in African catfish need to be elucidated. This study examined the effects of dietary *β*-mannanase on the growth performance, antioxidant capacity, liver enzymes, immunity, and protection of African catfish, *C. gariepinus*, against *A. hydrophila* infection.

## 2. Materials and Methods

### 2.1. Preparation of Experimental Diets and Fish Culture Regime

Five diets with equal levels of nitrogen were formulated to contain 400 g/kg of crude protein (CP) and were enriched with different concentrations of *β*-mannanase at 0, 1500, 3000, 4500, or 6000 thermostable endo, 1,4-*β*-mannanase units (TMUs)/kg (TMU = one mannanase unit) of diet, as detailed in Table 1. The diets were homogenized with 100 mL/kg of distilled water and then pelleted (2.0 mm) using a pelleting machine (Model HSDGP-60, Zhengzhou E.P. Machinery, Zhengzhou, China). The resulting dried diets were stored at −20°C until they were needed. Furthermore, the diets were reformulated every 2 weeks to ensure that nutrients were preserved. The *β*-mannanase 8000 TMU/g was obtained from Natupulse TS, BASF, Ludwigshafen, Germany, while other components were procured from local sources. Juvenile African catfish (*C. gariepinus*), with an average weight of 12.1 ± 0.1 g, were obtained from a reputable farm and placed in 1 m^3^ plastic tanks. Over a 2-week period, the fish were fed a commercial diet containing 400 g/kg of CP. Subsequently, the 300 fish were randomly allocated into 20 tanks, each with a capacity of 100 L, using a completely randomized design. Over the 12-week period, 20 fish per tank were fed experimental diets three times daily, at 5-h intervals, from 7:00 to 17:00, until they were satiated. The tanks were equipped with aerators and supplied with flowing water from an oxygenated storage tank. A mercury-in-glass thermometer, a digital dissolved oxygen (DO) meter (AVI-660, Labtech International Ltd., Heathfield, United Kingdom), and a digital pH meter (Photoic 20, Labtech International Ltd., Heathfield, United Kingdom) were used to measure water temperature, pH, and DO levels twice a day. The pH readings ranged from 7.6 to 7.8, while the DO levels varied from 5.5 to 6.0 mg/L, and the water temperature fluctuated between 28.5 and 29.2°C. Notably, all recorded values for pH, DO, and temperature were found to be within the recommended parameters [29]. After a 12-week feeding study, the fish from every tank were gathered and weighed collectively. The following formulas were used to quantify feed utilization and assess growth performance:

  
$$\begin{array}{c} \text{Weight gain }\left(\text{g}\right)=\text{Final weight }\left(\text{g}\right)\text{–initial weight }\left(\text{g}\right), \end{array}$$

  
$$\begin{array}{c} \text{Weight gain }\left(\%\right)=100\times \left(\frac{\text{final weight }\left(\text{g}\right)\text{–initial weight }\left(\text{g}\right)}{\text{initial weight }\left(\text{g}\right)}\right), \end{array}$$

  
$$\begin{array}{c} \text{Specific growth rate }\left(\text{SGR};\;\%/\text{day}\right)\;=\;100\;\left[\text{LN final body weight }\left(g\right)\;-\text{LN initial body weight }\left(g\right)\right]/\text{Experimental period}, \end{array}$$

 
$$\begin{array}{c} \text{Feed intake}=\text{The total weight of feed consumed }\left(\text{g}\right)\text{ by the fish throughout the experimental period}, \end{array}$$

  
$$\begin{array}{c} \text{Feed conversion ratio }\left(\text{FCR}\right)=\frac{\text{Feed intake }\left(\text{g}\right)}{\text{Weight gain }\left(\text{g}\right)}, \end{array}$$

  
$$\begin{array}{c} \text{Fish survival }\left(\%\right)=100\times \left(\frac{\text{final number of fish}}{\text{initial number of fish}}\right). \end{array}$$

### 2.2. Digestive Enzyme Analysis

An investigation into the digestive enzymes of fish was conducted by analyzing the gastrointestinal tracts (GIT) of fish that were fed experimental diets. The GIT of three fish from each experimental group was homogenized at a ratio of one part GIT to 10 parts deionized water, followed by centrifugation at 5000 g for 15 min at 4°C. Chymotrypsin, trypsin, lipase, protease, and *α*-amylase levels were measured by adding portions to the separated supernatants in Eppendorf tubes [30]. In brief, Azocasein (2%) in Tris-HCl with a pH of 7.5 was used as a substrate. The enzyme activity was quantified as the release of 1 μmol of n-nitrophenol per mg of protein per minute. Lipase activity was measured using a substrate mixture containing 0.53 mM p-nitrophenyl myristate, 0.25 mM 2-methoxy ethanol, 5 mM sodium cholate, and 0.25 M Tris-HCl (pH 9.0). This mixture (0.5 mL) was incubated at 30°C for 15 min. The reaction was halted by adding 0.7 mL of an acetone/n-heptane solution (5:2, v/v). After vigorous shaking, the mixture was centrifuged at 6080 g for 2 min, and the optical density of the aqueous phase was recorded at 405 nm. The enzyme activity was expressed as the release of 1 μmol of n-nitrophenol per mg of protein per minute. The crude enzyme extract was incubated with a 1% (w/v) starch solution in 0.02 M sodium phosphate buffer containing 0.006 M NaCl (pH 6.9) for 4 min at 25°C. Subsequently, 0.5 mL of 1% (w/v) dinitrosalicylic acid (DNS) solution was added to the reaction mixture, which was then boiled for 5 min and cooled to room temperature. The optical density was measured after adding 5 mL of distilled water to the final mixture, and the specific activity of *α*-amylase was expressed as micromole of maltose produced per milligram of protein per minute at 25°C [31].

### 2.3. Intestinal Morphometry

Three fish were anesthetized using a concentration of 30 mg/L of buffered tricaine methane sulfonate. Subsequently, the fish underwent dissection, and the intestines and livers were extracted for the purpose of measuring gut morphometry. The liver and intestine tissues were preserved in 20% neutral buffered formalin (Sigma–Aldrich, St. Louis, MO, United States). Typically, these samples were sectioned into 5-μm slices, embedded in paraffin wax for histological analysis, and stained with hematoxylin and eosin (Sigma–Aldrich). The width, length, and depth of the villi were measured using a micrometer and an Olympus CX21 light microscope (Tokyo, Japan) [32].

### 2.4. Digesta pH and Viscosity

A pH meter (Photoic 20, Labtech International Ltd., Heathfield, United Kingdom) inserted straight into the intestinal digesta was used to measure the pH of the digesta. To obtain the liquid phase, faeces samples were centrifuged for 10 min at 3000 rpm × *g* (SKU: 919101-03, 2273 Granite Way, Suite A, Laguna Hills, Califonia, United States). With the temperature set to 28°C, the produced supernatant was added to a RaeSung Digital Rotary Viscometer Viscosity Tester Meter (NDJ-9S, RaeSung Inc., India) The average 50.0/s shear rate was used to assess viscosity, and the apparent viscosity in centipoise (cP) was used to record the viscosity data [33].

### 2.5. Evaluation of Hematological and Biochemical Indices

At the conclusion of the growth study, the fish were subjected to a 24-h fasting period and then anesthetized for 5 min using sodium bicarbonate-buffered tricaine methanesulfonate (MS222, 30 mg/L, Syndel, Ferdale, Washington, United States). Following this, blood samples were obtained from the caudal veins of three fish in each tank using a needle and syringe. The blood sample was divided into two portions. The first portion was placed in anticoagulant bottles containing lithium heparin and left at room temperature. Hemoglobin (Hb) levels were measured using the Vankampen and Ziglstra [34] method, while hematocrit (Ht), red blood cell (RBC), white blood cell (WBC), and platelet counts were assessed using the Brown's [35] method. Furthermore, the Wright–Giemsa stain method determined the differential counts of lymphocytes, heterophils, monocytes, eosinophils, and basophils. Conversely, the serum was isolated by centrifuging the clotted second portion of the blood at 5000 × *g* for 10 min at 35°C. Subsequently, the levels of alanine aminotransferase (ALT), aspartate aminotransferase (AST), and alkaline phosphatase (ALP) were measured using the colorimetric method with Randox commercial kits (Randox Laboratories Ltd. Crumlin, County Antrim, United Kingdom) [36]. Additionally, glucose levels were measured colorimetrically following the method described by Trinder [37]. Allain et al.'s [38] method calculated cholesterol, while total protein and albumin were assessed [39].

### 2.6. Determination of Antioxidant and Immune Parameters

Using diagnostic reagent sets provided by Randox Laboratories in Crumlin, County Antrim, United Kingdom, the samples underwent centrifugation at 10,000 × *g* for 20 min at 4°C to assess the activity of the enzyme glutathione peroxidase (GPx) (Cat. No.: RS504; No.: CT211; Detection limit: 400–410 nm; optimal wavelength: 205 nm); and superoxide dismutase (SOD) (Cat. No.: SD124). The activities of reduced glutathione (GSH), glutamate cysteine ligase (GCL), and glutathione-S-transferase (GST) were assessed by conjugating 1 mM of GSH with 1 mM of 1-chloro-2,4-dinitrobenzene (CDNB) and measuring the absorbance at 340 nm. Agar wells were created on a circular reaction plate, and 25 L aliquots of the GCL reaction mixture were added to each well. Additionally, 25 L of 2 mM cysteine, previously dissolved in a buffer, was transferred into the reaction plate using pipettes. Furthermore, serum lysozyme and respiratory burst activities were assessed using the colorimetric method described by Henry [39] and the nitroblue tetrazolium dye method as outlined by Secombes [40]. The samples were added to enzyme assay buffers, homogenized at 4°C for 10 min, and then centrifuged at 10,000 × *g* for 10 min at 4°C to collect the supernatants for analyses. The enzyme activities were determined based on the protein concentration. Specifically, the protease activity (U/mg protein) was measured using the azocasein hydrolysis assay, while the esterase activity (U/mg protein) was determined according to the method described [41].

### 2.7. Bacterial Challenge Test

The *A. hydrophila* (American type culture collection [ATCC] 4356) strain was cultured in nutrient broth at 30°C for 24 h, then centrifuged and resuspended in 1.0 mL of 0.1% peptone water to determine the LD_50_ [42, 43]. The trial-and-error method was used to determine the highest dose that resulted in 0% mortality and the lowest dose that caused 100% fatality. Mortality rates were evaluated for five doses covering the spectrum from the least to the most tolerated levels. Correction factors were calculated using the formula: 0% mortality = 100 × (0.25/*n*) and 100% mortality = 100 × (*n*−0.25)/*n*, where *n* represents the total number of deaths. Probit units were obtained from the probit database to convert percentage mortality to probit values. The LD50 value was determined by plotting the probit value against log doses and identifying the dose corresponding to probit 5. Subsequently, 10 fish from each tank were intraperitoneally injected with 0.1 mL of *A. hydrophila* (5.0 × 10^5^ CFU/mL), and feeding was resumed after 24 h. The fish were monitored for 14 days to detect any signs of mortality. Mortalities rate was estimated according to mortalities rate (%) = number of dead fish/number of infected fish × 100.

### 2.8. Statistical Analysis

The collected data were assessed for homogeneity of variance and normality of distribution using the Kolmogorov–Smirnov and Bartlett tests. Subsequently, a one-way analysis of variance was used for data analysis. Orthogonal comparisons (linear and quadratic contrasts) were conducted at a significance level of 5% using SPSS Version 20 to statistically evaluate the growth performance, antioxidant capacity, liver enzymes, immunological responses, and survival of African catfish in response to *A. hydrophila* infection. The methodology outlined by Dytham [44] was followed for the analysis.

## 3. Results

### 3.1. Growth Performance

The growth performance of African catfish significantly improved (*p*  < 0.05) with the inclusion of dietary *β*-mannanase levels. Fish-fed diets supplemented with *β*-mannanase showed significantly higher final weight (g), weight growth (g), and weight gain (%). Furthermore, fish fed with feed fortified with *β*-mannanase showed significantly higher specific growth rates (SGRs) compared to those fed with the control diet. Furthermore, it was observed that fish-consuming diets containing *β*-mannanase showed increased feed intake and a significantly higher hepatosomatic index (*p*  < 0.05) compared to fish fed the control diet (Table 2). Conversely, adding *β*-mannanase to the diet led to a significant decrease in the feed conversion ratio compared to a diet consisting solely of fish feed. However, there was no noticeable effect of dietary *β*-mannanase levels on fish survival (Table 2). The relationships between final weight (g) and *β*-mannanase levels (Equation 1), body weight gain (g) and *β*-mannanase levels (Equation 2), SGR (%/day) and *β*-mannanase levels (Equation 3), feed intake (g) and *β*-mannanase levels (Equation 4), feed conversion rate (FCR) and *β*-mannanase levels (Equation 5), and hepatosomatic index (%) and *β*-mannanase levels (Equation 6) were best described by the second-order polynomial regression:

(1) $$\begin{array}{c} \text{y}=-2\text{E}-06{\text{x}}^{2}+0.0169\text{x}+95.6;{\text{R}}^{2}=0.9609, \end{array}$$

(2) $$\begin{array}{c} \text{y}=-2\text{E}-06{\text{x}}^{2}+0.0169\text{x}+83.366;{\text{R}}^{2}=0.9613, \end{array}$$

(3) $$\begin{array}{c} \text{y}=-3\text{E}-08{\text{x}}^{2}+0.0003\text{x}+3.6783;{\text{R}}^{2}=0.9668, \end{array}$$

(4) $$\begin{array}{c} \text{y}=-4\text{E}-07{\text{x}}^{2}+0.008\text{x}+111.35;{\text{R}}^{2}=0.8648, \end{array}$$

(5) $$\begin{array}{c} \text{y}=2\text{E}-08{\text{x}}^{2}-0.0002\text{x}+1.3657;{\text{R}}^{2}=0.8571, \end{array}$$

(6) $$\begin{array}{c} \text{y}=-6\text{E}-09{\text{x}}^{2}+5\text{E}-05\text{x}+5.2434;{\text{R}}^{2}=0.9479. \end{array}$$

### 3.2. Digestive Enzymes, Gut Mpphormetry, and Digesta SCFAs, Viscosity, and pH of African Catfish Juveniles Fed the Experimental Diets

Table 3 illustrates the digestive enzyme activity of African catfish when they were fed diets supplemented with *β*-mannanase. The levels of amylase, protease, and lipase were significantly higher in fish fed with *β*-mannanase-fortified diets compared to the control group (*p*  < 0.05). However, the activities of chymotrypsin and trypsin were not significantly affected (*p*  > 0.05). Furthermore, the incorporation of dietary *β*-mannanase led to a significant improvement in gut histomorphometric parameters. Specifically, the length and width of intestinal villi, as well as absorption, were significantly increased, while crypt depth decreased in African catfish compared to those fed the basal diet (*p*  < 0.05) (Table 4).  Table 5 presents the impact of *β*-mannanase supplementation on the production of SCFAs in the gut, as well as pH and viscosity values. Acetic acid and butyric acid levels in the gut increased in a dose-dependent manner with the addition of *β*-mannanase supplementation. Conversely, propionic acid and pH were not significantly affected (*p*  > 0.05) by increasing *β*-mannanase supplementation, while gut viscosity decreased in both a linear and quadratic manner (*p*  < 0.05).

### 3.3. Haematological and Plasma Biochemistry Profiles of African Catfish Juveniles Fed the Experimental Diets

**Figure 1:**
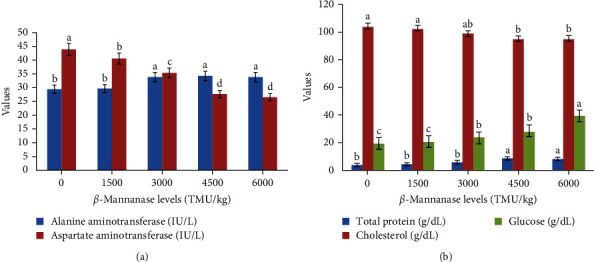
Liver enzymes (A) and plasma biochemistry (B) activities of African catfish fed diets supplemented with different levels of *β*-mannanase for 12 weeks. Bars with the same colour having the same letter in the same row are not significantly different at *p* > 0.05.

Table 6 presents the hematological profiles of African catfish that were fed different doses of *β*-mannanase. The fish that were fed diets fortified with *β*-mannanase showed significantly higher hematological parameters (*p*  < 0.05) compared to the control group. The levels of hematological indices, such as Ht, Hb, RBCs, WBCs, and platelets, exhibited a significant dose-dependent increase. Furthermore, the incorporation of *β*-mannanase in the African catfish diet resulted in a decrease in the levels of lymphocytes, heterophils, monocytes, eosinophils, and basophils (*p*  < 0.05; Table 6). Furthermore, the administration of *β*-mannanase to African catfish resulted in a significant reduction in cholesterol and ALT levels, accompanied by significant increases in AST, glucose, and total protein levels (Figure 1) (*p*  < 0.05). When compared to the control group, fish that were fed diets containing 4500–6000 TMU/kg showed the lowest levels of cholesterol and ALT, and the highest levels of AST, glucose, and total protein.

### 3.4. Antioxidant Activities of the Fish

Table 7 illustrates that the addition of *β*-mannanase to the diets led to increased levels of GPx, GST, GSH, SOD, and GCL, and decreased malondialdehyde (MDA) values in African catfish. These differences were found to be statistically significant (*p*  < 0.05). Specifically, the fish fed a diet containing 6000 mg/kg of *β*-mannanase TMU/kg exhibited the highest levels of GPx, GST, GSH, SOD, and GCL, while the control group showed the lowest levels. Conversely, the MDA levels were lowest in the fish fed the diet containing 6000 TMU/kg of *β*-mannanase, and highest in those fed the control diet.

### 3.5. Immunity Response and Postchallenge Mortalities of African Catfish

African catfish that were fed diets containing *β*-mannanase showed a significantly higher nonspecific immune response compared to the control group (Table 8). This was indicated by increased levels of lysozyme, respiratory burst, proteases, and esterase activity. Specifically, those fed with feed fortified with 6000 TMU of *β*-mannanase/kg diet demonstrated the highest levels of immune response (Table 8). Furthermore, the mortality rate of African catfish following *A. hydrophila* infection was significantly reduced (*p*  < 0.05) in the groups that received *β*-mannanase supplementation compared to the control group. The control diet group had the highest mortality rate (52.5%) (Table 8), while the group fed with 6000 TMU *β*-mannanase/kg diet exhibited the lowest mortality rate (7.5%), with an optimal inclusion level of 5800 TMU *β*-mannanase/kg diet (*p*  < 0.05).

## 4. Discussion

The results of the current study showed that giving African catfish supplements of *β*-mannanase improved their growth and nutrient uptake. This study's results align with those of Chen et al. [14], Dawood and Shi [17], Sallam et al. [45], and da Cruz et al. [22] who discovered that feeding common carp, marbled spinefoot rabbitfish, and Nile tilapia a diet enhanced with *β*-mannanase caused them to grow greater weight. In contrast to the results of da Cruz et al. [22], feeding African catfish with *β*-mannanase-based meals that include more than 4500 TMU/kg lowered the fish's body weight gain. The disparities noted in this study may be ascribed to the Nile tilapia's ability to efficiently utilize a plant-based diet in contrast to the African catfish. The study's findings of increased feed intake and relatively lower FCR provide additional evidence for the remarkable growth performance observed. Notably, the addition of *β*-mannanase is linked to higher energy expenditure, resulting in increased growth rates and greater feed consumption [22].

The addition of *β*-mannanase to the diet of African catfish elicited digestive enzyme activity. The supplementation of *β*-mannanase in African catfish elicited higher levels of *α*-amylase, protease, and lipase, indicating the digestive tract's capacity to digest feed and its metabolic functionality. This concurrently increased the observed growth. Comparable findings were noted for Nile tilapia [22, 45] and common carp [17]. Studies on intestinal histology could be used to assess the nutritional physiological mechanisms behind the growth-promoting effects of *β*-mannanase feed additions in cultured fish. The current study's findings concur with earlier reports on increased villi length, width, and absorption area in the guts of Nile tilapia [22] and Common carp [17]. The increased height of the villi and expanded absorptive area observed in the African catfish in this study were anticipated to enhance nutrient uptake, utilization, and growth. This is because the digestive and absorptive processes for nutrients primarily occur in the intestines of fish [46–48]. The enlarged crypts in the treated animals would have stimulated the release of electrolytes by cryptal cells, thereby facilitating water secretion into the intestinal lumen for digestion and nutritional absorption [49]. Conversely, the decreased depth of the crypts in this study was associated with more effective nutrient absorption. The reduction in cryptal depth accelerated the slower turnover rate of the intestinal epithelium, resulting in increased nutritional intake and growth, and reduced maintenance demands for the animals [46].

The current investigation revealed that dietary *β*-mannanase significantly raised the levels of butyric and acetic acids. The synthesis of SCFAs predominantly occurs through the endogenous fermentation of carbohydrates by the gut bacteria [28, 50, 51] has indicated that the composition of fermentable carbohydrates significantly influences the profile of SCFAs, thereby affecting gut microbial activity. The presence of SCFAs has been linked to positive outcomes such as improved growth performance, feed efficiency, immune response, survival rate, and enhanced intestinal morphology [52–56].

Our findings show that *β*-mannanase directly affects the synthesis of SCFAs, as demonstrated by the rise in acetic and butyric acid levels in response to elevated dietary *β*-mannanase levels. One of the SCFAs, butyric acid, is usually used (99%) to modify the junction proteins between intestinal cells, hence improving the permeability and epithelial barrier. Improvements in gut health are only attributable to this protective effect on the intestinal mucosa and the rise in villus density [57, 58]. The synthesis of SCFAs affects intestinal shape and pH as well; it is anticipated that the digestion of NSPs will lower intestinal pH. Because it can prevent the growth of Gram-negative bacteria, which are predominantly linked to diseases, a lower pH in the intestine is important for regulating the microbiota and lowering the number of pathogenic organisms [59]. The results of this investigation agree with the findings of da Cruz et al.'s [22] study.

Hematological and biochemical markers are commonly used to assess the health of fish [4]. The fish in the current study showed significantly higher levels of Ht, Hb, RBCs, WBCs, and platelets when given a diet enriched with *β*-mannanase, compared to those fed a standard diet. These findings also indicated that the blood function of the African catfish was improved by *β*-mannanase. The concentration of Hb, RBCs, and Ht in the blood affects tissue oxygenation. As a result, the enhanced tissue oxygenation in African catfish that were fed *β*-mannanase was linked to elevated levels of Ht, RBCs, Hb, and lymphocytes. Erythrocytes provide important information about the overall health of fish, whereas WBCs fight foreign diseases through phagocytic activities. However, the numbers of monocytes, eosinophils, basophils, and heteroblasts were decreased by the *β*-mannanase supplementation. The results of this investigation align with the findings of El-Dakar et al.'s [47] study, who documented elevated levels of haematological variables in Nile tilapia given *β*-mannanase as a diet. Fish biochemical profiles offer vital information about the health of the fish. In this study, the reduction in the neutrophils, monoctyes, and eosinophils levels in fish-fed dietary *β*-mannanase revealed that fish were not stressed and could be linked to an enhanced immunity. Macrophages play a vital role in detecting and engulfing pathogens and dead cells, and in producing inflammatory cytokines to signal other immune cells. Similarly, eosinophils and basophils are involved in the defense against foreign infections and in allergic responses. They release inflammatory mediators that help modulate the innate immune response. Furthermore, significantly higher platelets counts in fish-fed experimental diets than the control group indicate a better protection. Platelets contribute to innate immunity by releasing antimicrobial peptides and inflammatory mediators, and by interacting with immune cells to enhance pathogen clearance which is supported by the result of the *Aeromonas hydrophila* challenge tests in this study. Furthermore, there was a substantial increase in AST, glucose, and total protein levels; conversely, there was a significant drop in cholesterol and ALT levels with elevated dietary *β*-mannanase levels. Similar studies [17, 22] reported that adding *β*-mannanase to the diets of common carp and Nile tilapia resulted in lower plasma parameter levels.

Other important enzymes such as GPx, GST, SOD, and GCL play a significant role in reducing oxidative stress in fish. Ameur et al. [60] and Ming et al. [61] argued that elevated levels of heavy metals, bacteria, and toxins can trigger oxidative stress, resulting in a decrease in the antioxidant defense system. This phenomenon represents a significant source of stress for fish raised in aquaculture environments [62, 63]. The present study's findings demonstrated that fish given *β*-mannanase had significantly increased levels of GPx, GST, GSH, SOD, and GCL, indicating that the enzyme may enhance the fish's capacity for antioxidant defence. Conversely, the fish that were given feed supplemented with *β*-mannanase exhibited significantly reduced MDA levels, suggesting that *β*-mannanase could potentially mitigate the intensity of lipid peroxidation.

Because it is the first line of defence against a wide range of dangerous pathogens, fish's innate immune system is essential for preserving fish health [64–66]. Lysozyme, a vital component of fish's innate defence, is found in large quantities in fish mucus and blood and is capable of quickly scavenging invasive pathogens and germs [67, 68]. The vertebrate complement system is crucial for immunity, and proteases, regulators, pattern-recognition molecules, and cell surface receptors are some of the elements required to fend off infections [4, 67–74]. The addition of dietary *β*-mannanase in the current investigation raised the levels of lysozyme and respiratory burst activities, especially at the highest values of 4500–6000 TMU/kg diet. Protease and esterase are also significant factors to take into account when predicting immunoreactions. The levels of protease and esterase in fish-fed *β*-mannanase-based diets were significantly greater than in fish fed a control diet, indicating that dietary *β*-mannanase could enhance fish immunity.

Furthermore, boosting an organism's immune system may result in an improved ability to defend itself against bacterial and other infections, thereby fostering the healthy growth of the animals [4, 5, 74]. Therefore, it is crucial to enhance the immunity, anti-inflammatory, and antioxidant capabilities of fish for their overall well-being. In the present study, after a 2-week challenge with *A. hydrophila*, the groups that were supplemented with *β*-mannanase showed a significantly higher survival rate among African catfish. Similar studies have shown the beneficial effects of *β*-mannanase supplementation on fish immunity. This is supported by a notable increase in lysozyme activity, respiratory burst activity, and phagocytic activity in common carp and Nile tilapia [14, 17]. These findings suggested that the mannan hydrolyzed product is indirectly inducing an immunological response. In general, mannan oligosaccharides function as prebiotics, stimulate the growth of probiotics such as Lactobacillus and Bifidobacterium species, inhibit the colonization of pathogens in the gut by binding with mannose-specific type-I fimbriae, and activate the immune system by activating protein and pattern-recognition receptors. The enhanced immunity and antioxidant properties of *β*-mannanase cannot be separated from this elevated bacterial resistance.

## 5. Conclusion

The results of the study show that feeding African catfish, *Clarias gariepinus*, *β*-mannanase enhanced growth performance, increased activity of digestive enzymes, gut morphology, enhanced generation of SCFAs, digesta pH, and improved antioxidant profiles and immunity at the optimum dose of 5800 TMU/kg diet. Additionally, *β*-mannanase shielded African catfish from dying from an *A. hydrophila* infection. According to these findings, feeding African catfish with *β*-mannanase increases the nutritional content of sustainable plant-based feeds used in African catfish rearing.

## Figures and Tables

**Table 1:** Ingredient composition of the control diet (g/kg diet).

Ingredients	*β*-Mannanase levels (TMU/kg)				
	0 (control)	1500	3000	4500	6000
Fish meal (55% CP)	290	290	290	290	290
Soybean meal (43% CP)	290	290	290	290	290
Groundnut cake (45% CP)	230	230	230	230	230
Yellow maize	150	150	150	150	150
Vegetable oil	15	15	15	15	15
Vitamins and minerals premix^a^	15	15	15	15	15
Starch	10	10	10	10	10
Total	1000	1000	1000	1000	1000
*β*-Mannanase levels (TMU/kg)	0.0	1500	3000	4500	6000
Proximate chemical analysis (g/kg)					
Dry matter	95	95	93	83	93
Crude protein	379	379	379	379	379
Ether extract	180	182	182	181	181
Total ash	204	204	203	204	204
Crude fiber	59	60	58	60	60

Abbreviations: CP, crude protein; TMU, thermostable endo, 1,4-*β*-mannanase unit.

^a^Premixes, HI-MIXAQUA (fish) each 1 kg contains as follows: Vitamin A, 4,000,000 International Unit (IU); vitamin D3, 800,000 IU; vitamin E, 40,000 IU; vitamin K3, 1600 mg; vitamin B1, 4000 mg; vitamin B2, 3000 mg; vitamin B6, 3800 mg; vitamin B12, 3 mcg; nicotinic acid, 18,000 mg; pantothenic acid, 8000 mg; folic acid, 800 mg; biotin, 100 mcg; choline chloride, 120,000 mg; iron, 8000 mg; copper, 800 mg; manganese, 6000 mg; zinc, 20,000 mg; iodine, 400 mg; selenium, 40 mg; vitamin C (coated), 60,000 mg; inositol, 10,000 mg; cobalt, 150 mg; lysine, 10,000 mg; methionine, 10,000 mg; antioxidant, 25,000 mg.

**Table 2:** Growth performance of African catfish juveniles fed the experimental diets for 12 weeks.

Parameters	*β*-Mannanase levels (TMU/kg)					SEM	*p*-Values	
	0	1500	3000	4500	6000		Linear	Quadratic
Initial body weight (g)	12.2	12.3	12.2	12.2	12.3	0.021	0.515	0.636
Final body weight (g)	94.5^d^	121.3^c^	126.5^b^	142.3^a^	138.6^a^	5.235	0.001	<0.001
Body weight gain (g)	82.3^e^	109.0^d^	114.3^c^	130.1^a^	126.3^b^	3.018	<0.001	<0.001
Body weight gain (%)	674.6^e^	886.2^d^	936.9^c^	1066.4^a^	1026.8^b^	8.117	<0.001	<0.001
SGR (%/day)	3.66^d^	4.09^c^	4.18^c^	4.39^a^	4.32^b^	0.105	<0.001	<0.001
Feed intake (g)	113.4^d^	119.6^c^	127.4^b^	146.9^a^	140.7^a^	1.282	<0.001	<0.001
FCR	1.4^a^	1.1^b^	1.1^b^	1.1^b^	1.1^b^	0.101	0.041	0.003
Hepatosomatic index (%)	5.24^c^	5.32^b^	5.33^b^	5.36^a^	5.33^b^	0.329	0.005	0.001
Survival (%)	97.5	98.75	97.5	98.75	98.75	0.023	0.822	0.517

*Note:* Means within a row with different superscripts are significantly different (*p*  < 0.05).

Abbreviations: FCR, feed conversion ratio; SEM, pooled standard error of the means; SGR, specific growth rate; TMU, thermostable endo, 1,4-*β*-mannanase unit.

**Table 3:** Digestive enzymes in the gut of African catfish juveniles fed the experimental diets for 12 weeks.

Parameters (U/mg protein)	*β*-Mannanase levels (TMU/kg)					SEM	*p*-Values	
	0	1500	3000	4500	6000		Linear	Quadratic
Chymotrypsin	0.14	0.15	0.15	0.15	0.14	0.001	0.511	0.713
Trypsin	0.15	0.15	0.15	0.15	0.15	0.001	0.223	0.597
Amylase	0.26^b^	0.26^b^	0.37^a^	0.39^a^	0.38^a^	0.011	0.001	0.001
Protease	0.27^c^	0.29^bc^	0.32^b^	0.35^a^	0.34^a^	0.014	<0.001	<0.001
Lipase	0.32^c^	0.36^b^	0.39^b^	0.45^a^	0.44^a^	0.010	<0.001	<0.001

*Note:* Means within a row with different superscripts are significantly different (*p*  < 0.05).

Abbreviations: SEM, pooled standard error of the means; TMU, thermostable endo, 1,4-*β*-mannanase unit.

**Table 4:** Gut morphometry of African catfish juveniles fed the experimental diets.

Parameters (U/mg protein)	*β*-Mannanase levels (TMU/kg)					SEM	*p*-Values	
	0	1500	3000	4500	6000		Linear	Quadratic
Total villus height (µm)	276.4^d^	303.6^c^	342.2^b^	368.0^a^	400.2^a^	11.864	<0.001	<0.001
Villus width (µm)	125.1^e^	134.8^d^	148.2^c^	163.7^b^	183.1^a^	6.142	0.001	0.0132
Villus height:villus width	2.2	2.3	2.3	2.2	2.2	0.011	0.142	0.327
Areas of absorption (µm^2^)	34,577.6^e^	40,925.3^d^	50,714.0^c^	60,241.6^b^	73,276.6^a^	28.527	<0.001	<0.001
Cryptal depth (µm)	521.4^a^	404.2^b^	383.4^c^	361.5^d^	305.2^e^	5.905	0.001	<0.001

*Note:* Means within a row with different superscripts are significantly different (*p*  < 0.05).

Abbreviations: SEM, pooled standard error of the means; TMU, thermostable endo, 1,4-*β*-mannanase unit.

**Table 5:** Digesta short-chain fatty acids, viscosity, and pH of African catfish juveniles fed the experimental diets.

Parameters	*β*-Mannanase levels (TMU/kg)					SEM	*p*-Values	
	0	1500	3000	4500	6000		Linear	Quadratic
Acetic acid (nmol/L)	9.7^d^	10.4^c^	11.9^b^	13.5^a^	12.8^a^	1.452	<0.001	<0.001
Propionic acid (nmol/L)	1.1	1.1	1.2	1.2	1.1	0.010	0.236	0.114
Butyric acid (nmol/L)	0.4^c^	0.6^b^	0.9^a^	0.9^a^	0.9^a^	0.031	<0.001	<0.001
Viscosity (cP)	3.1^a^	2.8^b^	2.6^c^	2.5^c^	2.3^d^	0.087	<0.001	<0.001
pH	7.8	7.8	7.6	7.5	7.4	0.211	0.512	0.611

*Note:* Means within a row with different superscripts are significantly different (*p*  < 0.05).

Abbreviations: cP, centipoise; SEM, pooled standard error of the means; TMU, thermostable endo, 1,4-*β*-mannanase unit.

**Table 6:** Hematological profiles of African catfish juveniles fed diets containing various levels of *β*-mannanase for 12 weeks.

Parameters	*β*-Mannanase levels (TMU/kg)					SEM	*p*-Values	
	0	1500	3000	4500	6000		Linear	Quadratic
Hematocrits (%)	23.15^c^	35.22^b^	36.87^b^	39.52^a^	39.52^a^	2.011	<0.001	<0.001
Hemoglobin (g/dL)	7.71^d^	10.64^c^	11.12^c^	13.42^b^	14.17^a^	1.031	0.001	<0.001
Red blood cell (×10^6^/μL)	1.39^d^	3.14^c^	3.29^b^	3.57^a^	3.96^a^	0.120	<0.001	<0.001
White blood cell (×10^3^/μL)	105.06^d^	162.51^c^	183.18^b^	186.43^a^	193.27^a^	7.892	<0.001	<0.001
Platelets (×10^6^/μL)	201.25^d^	231.01^c^	273.51^b^	288.16^a^	296.22^a^	11.023	<0.001	<0.001
Lymphocytes (%)	58.35^c^	61.32^b^	62.51^b^	63.85^ab^	64.55^a^	5.101	<0.001	<0.001
Heterocytes (%)	31.58^a^	29.32^ab^	28.47^b^	27.76^b^	27.23^b^	2.258	<0.001	<0.001
Monocytes (%)	3.63^a^	3.46^ab^	3.35^b^	3.11^c^	3.14^c^	0.162	0.001	<0.001
Eosinophils (%)	4.99^a^	4.52^a^	4.38^b^	4.15^c^	4.02^c^	0.189	0.001	0.001
Basophils (%)	1.45^a^	1.38^b^	1.29^c^	1.13^c^	1.06^c^	0.010	0.021	0.001

*Note:* Means within a row with different superscripts are significantly different (*p*  < 0.05).

Abbreviations: SEM, pooled standard error of the means; TMU, thermostable endo, 1,4-*β*-mannanase unit.

**Table 7:** Changes in antioxidant profiles of African catfish juveniles fed diets containing various levels of *β*-mannanase for 12 weeks.

Parameters	*β*-Mannanase levels (TMU/kg)					SEM	*p*-Values	
	0	1500	3000	4500	6000		Linear	Quadratic
GPx (IU/L)	45.53^c^	56.11^b^	62.35^a^	70.33^a^	71.23^a^	2.514	0.002	<0.001
GST (nmol/L)	31.21^c^	36.49^b^	42.27^a^	43.13^a^	43.52^a^	2.239	<0.001	<0.001
Reduced GSH (nmol/L)	54.23^d^	54.86^d^	64.37^c^	70.26^b^	76.23^a^	5.137	<0.001	<0.001
MDA (µmol/mg protein)	5.21^a^	4.36^b^	3.32^c^	2.72^d^	2.31^e^	0.203	0.001	<0.001
SOD (IU/L)	21.16^d^	26.54^c^	31.22^b^	36.43^b^	39.21^a^	1.313	<0.001	<0.001
GCL (nmol/L)	27.26^d^	30.34^c^	35.99^b^	41.67^a^	42.41^a^	2.061	<0.001	<0.001

*Note:* Means within a row with different superscripts are significantly different (*p*  < 0.05).

Abbreviations: GCL, glutamate cysteine ligase; GPx, glutathione peroxidase; GSH, glutathione; GST, glutathione-S-transferase; MDA, malondialdehyde; SEM, pooled standard error of the means; SOD, superioxide dismutase; TMU, thermostable endo, 1,4-*β*-mannanase unit.

**Table 8:** Immune response and postinfection of African catfish juveniles fed diets containing various levels of *β*-mannanase for 12 weeks.

Parameters	*β*-Mannanase levels (TMU/kg)					SEM	*p*-Values	
	0	1500	3000	4500	6000		Linear	Quadratic
Lysozyme activity (unit/mg protein)	7.35^d^	11.45^c^	15.48^b^	19.62^a^	21.23^a^	2.334	0.001	<0.001
Respiratory burst activity (mg/mL)	0.32^c^	0.35^c^	0.48^b^	0.51^a^	0.55^a^	0.011	0.001	<0.001
Protease (U/mg protein)	40.24^d^	44.13^c^	50.33^b^	58.74^a^	58.98^a^	6.543	<0.001	<0.001
Esterase (U/mg protein)	25.32^d^	25.33^c^	39.51^b^	42.54^ab^	43.55^a^	6.417	<0.001	<0.001
Postchallenge mortalities (%)	52.5^a^	37.5^b^	20.0^c^	10.0^d^	7.5^d^	1.084	<0.001	<0.001

*Note:* Means within a row with different superscripts are significantly different (*p*  < 0.05).

Abbreviations: SEM, pooled standard error of the means; TMU, thermostable endo, 1,4-*β*-mannanase unit.

## Data Availability

Data are available upon request from the authors.
